# Intestinal flora of hepatitis C after direct antiviral drug therapy: A review

**DOI:** 10.1097/MD.0000000000042301

**Published:** 2025-08-01

**Authors:** Hui Li, Yang Liu, Ming Yu Lai

**Affiliations:** a Department of Gastroenterology, The First Affiliated Hospital of Guangxi Medical University, Nanning, China.

**Keywords:** chronic hepatitis C, direct antiviral drugs, intestinal flora, liver–gut axis, probiotics

## Abstract

Hepatitis C virus infection remains one of the most critical healthcare issues worldwide, attracting attention for its high chronicity and extrahepatic impact. The continued development of direct antiviral antivirals (DAAs) has made hepatitis C virus infection curable. In recent years, it has been found that dysbiosis is often observed in chronic liver diseases, and chronic hepatitis C (CHC) is one of them. However, the role of intestinal flora and whether DAA has any effect on it remains unclear. In our review, we summarize the changes in intestinal flora before and after DAA treatment in different stages of CHC, as well as the research progress of probiotics in recent years, to provide new thinking for further research on the interrelationship between intestinal flora and CHC as well as for the clinical treatment of CHC.

## 1. Introduction

**Figure 1.**
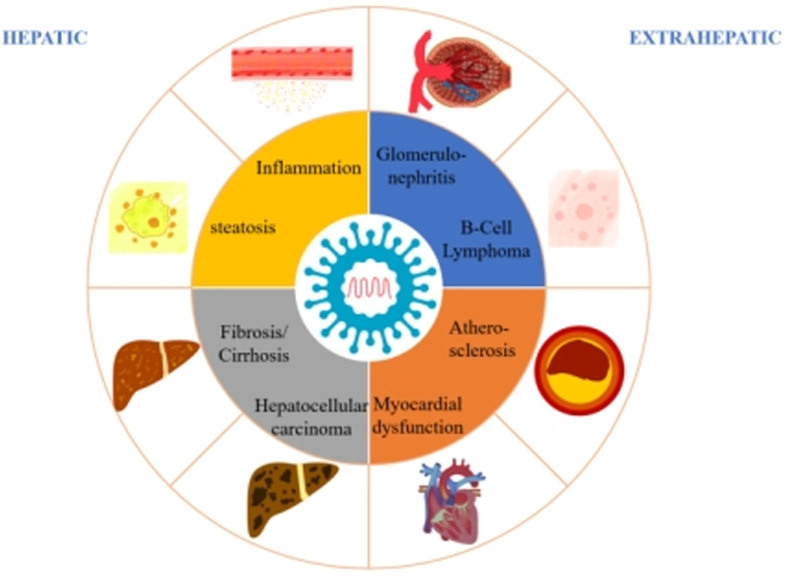
The principal hepatic and extrahepatic manifestations during CHC. CHC = chronic hepatitis C.

Chronic hepatitis C virus (HCV) infection remains a significant global health concern, impacting approximately 60 million individuals worldwide and potentially progressing to severe hepatic complications, including cirrhosis and hepatocellular carcinoma.^[1]^ Currently, direct-acting antivirals (DAAs) represent the preferred treatment option for patients infected with the HCV, achieving sustained virologic response (SVR) rates exceeding 95 percent.^[2]^ Studies have demonstrated that the eradication of HCV using DAAs is associated with a regression in liver fibrosis and a substantial reduction in the risk of cirrhosis and hepatocellular carcinoma. In addition, successful HCV clearance reduces a variety of extrahepatic manifestations, including diabetes, stroke, and chronic kidney disease.^[3]^ Recent data have also confirmed the survival advantage associated with HCV treatment, including in patients with mild liver disease.^[2]^ Figure 1 shows the principal hepatic and extrahepatic manifestations during chronic hepatitis C (CHC).

Gut microbial imbalance, characterized by disrupted microbial diversity and altered community structure, has emerged as a critical determinant in the pathogenesis and progression of multiple chronic hepatic disorders.^[4,5]^ Altered gut microbiota homeostasis, marked by disturbances in microbial diversity and community structure, significantly contributes to the disease progression of multiple chronic hepatic disorders.^[6]^ Studies have shown that intestinal microecological dysregulation exists even in mild chronic HCV infection, and these changes are more pronounced in progressive liver disease.^[7,8]^ In addition, bacterial translocation mediated by intestinal barrier impairment was observed in the absence of overt fibrosis or cirrhosis, indicating that compromised intestinal barrier integrity may precede hepatic fibrogenesis during the early pathogenesis of CHC virus infection.^[9,10]^ The importance of intestinal flora in HCV infection cannot be overstated. Table 1 shows the dysbiosis of intestine microbes in CHC.

**Table 1:** The dysbiosis of intestine microbes in CHC.

Dysbiosis of the microbes	Metabolite changes	References
Bacterioidetes↑	Lipopolysaccharide ↑	[7]
Enterobacteriaceae ↑	Deoxycholic acid ↓	
Viridans streptococci ↑	Lithocholic acid or ursodeoxycholic acid ↑	[11]
Firmicutes ↓		
Lactobacillus ↑		
Streptococcus ↑		[12]
Clostridiales ↓		

New evidence suggests that antiviral therapy may improve gut dysbiosis in patients who achieve SVR,^[13,14]^ particularly in those with early fibrosis at baseline.^[15]^ Clinical investigations have further demonstrated that nonclassical microbial translocation biomarkers, exemplified by lipopolysaccharide-binding protein, exhibit marked reductions following successful antiviral therapy for HCV infection.^[16]^ However, the profile of changes in the gut microbiota is inconsistent in the literature and influenced by multiple factors. Moreover, studies on the gut flora before and after DAA treatment for HCV infection are increasing; however, discrepancies exist in the findings.

In our paper, we systematically review the relevant studies on changes in intestinal flora before and after DAA treatment over the past few years, the research progress on probiotics in treating CHC, and the interrelationship between intestinal flora and CHC.

### 1.1. Intestinal flora and DAA therapy

Gut microecology is stabilized differently in different phases of the disease and is influenced by various factors. It has also been shown that the effect of treatment on the enterohepatic axis seems to depend directly on the stage of fibrosis and the time of assessment after treatment.^[17]^ Therefore, it is necessary to include cases with different disease levels and to analyze the influencing factors. Table 2 lists the relevant literature and main information.

**Table 2:** The relevant literatures and main information.

Author, year	Country	Follow-up time	Subjects	Conclusions
Pao-Yuan Huang, 2023	Prospective	Baseline/EOT 24	Healthy control/CHC with or without cirrhosis	The alpha-diversity indices Chao1 and ACE were not significantly different between the 3 groups. Simpson index was also not significantly different between groups. Subgroup analysis of patients with and without cirrhosis was performed for the CHC group. Microbial diversity was not significantly different among patients with and without cirrhosis who achieved SVR.
Natthaya Chuaypen, 2023	Prospective	EOT 72	Healthy control/HCV mono- and HCV/HIV coinfection	Among responders, significant restoration of alpha-diversity, BCoAT and LBP were observed in HCV patients with low-grade fibrosis (F0–F1), while HCV/HIV patients exhibited partial improvement at FUw72. I-FABP did not decline significantly in responders. Treatment induced microbiota changes with increasing abundance of SCFAs-producing bacteria, including Blautia, Fusicatenibacter, Subdoligranulum and Bifidobacterium.
Yao-Chun Hsu, 2022	Prospective	Baseline/EOT 12	CHC with or without cirrhosis (2 HBV dually infected patients)	The alpha-diversity of gut microbiome, as measured by observed species, Chao1 index, Shannon index, Simpson index, and inverted Simpson index, were significantly different between uninfected control and hepatitis C patients.
Takashi Honda, 2021	Prospective	Baseline/EOT/Post24	CHC without cirrhosis	The diversity of the gut microbiota did not significantly differ between Pre, EOT, and Post24.the relative abundances of Faecalibacterium and Bacillus increased at EOT, further increased at Post24, and were significantly increased at Post24 compared to Pre.
Natthaya Chuaypen, 2021	Prospective	Baseline/EOT 12	HCV mono- and HCV/HIV coinfection	Compared with HCV-mono-infected patients, HCV/HIV-coinfected individuals showed comparable microbial alpha-diversity but decreased Firmicutes–Bacteroidetes ratios. The improvement of microbial dysbiosis was observed in responders achieving sustained virological response across fibrosis stages but was not found in nonresponders. Responders with a low degree of fibrosis exhibited a recovery in alpha-diversity to levels comparable to those in healthy controls. Reciprocal alterations of increased beneficial bacteria and reduced pathogenic bacteria were also observed in responders.
Wellhöner et al, 2021	Prospective	Baseline/EOT 24/EOT 48	Healthy control/CHC with or without cirrhosis	The alpha-diversity increased numerical but not significantly from baseline to SVR24/48. When stratifying for the presence of liver cirrhosis, a significant increase in diversity was only seen in patients without cirrhosis. Differences in the microbial community structure induced by the achievement of SVR were only observed in patients without liver cirrhosis. In patients with liver cirrhosis and in the control group, no significant differences were observed.
Patricia Pérez-Matute, 2019	Prospective	Pre/EOT/Post12	CHC without cirrhotic	Neither the usage of DAAs nor 3 months in SVR are able to counteract the major changes induced by HCV in non-cirrhotic patients. Only mild improvements were observed in the abundance of Lachnospira and Dorea genera.
Francesca Romana Ponziani, 2018	Prospective	Pre/Post-1 yr	CHC with cirrhotic	The gut microbiota alpha-diversity in cirrhotic patients was significantly improved by the cure of HCV infection and a shift in the overall gut microbiota composition was observed compared to baseline. The abundance of potentially pathogenic bacteria (Enterobacteriaceae, Enterococcus, and Staphylococcus) was decreased after treatment. Although a significant reduction in the serum levels of cytokines and chemokines was observed post-DAA treatment, measures of intestinal permeability and inflammation remained unchanged.

CHC = chronic hepatitis C, DAAs = direct antiviral antivirals, HCV = hepatitis C virus, LBP = lipopolysaccharide-binding protein, SCFAs = short-chain fatty acids, SVR = sustained virologic response.

#### 1.1.1. Short and medium-term effects of DAA on gut microbiota in hepatitis C-infected patients

Some studies have shown that after achieving SVR with DAA therapy, the follow-up time was either 3 months or 6 months, and the diversity of the gut microbiota did not significantly change in patients with or without cirrhosis after HCV eradication.^[11,12,14]^ In line with these results, Chun Hsu et al’s study included some patients with concurrent HBV infection.^[18]^ However, their study was not specifically designed for patients with HBV and HCV-dual infection. Unlike previous studies, Chuaypen et al’s study recruited some patients with HCV/HIV coinfection. Comparative analyses revealed that HCV/HIV-coinfected individuals exhibited microbial α-diversity levels comparable to those with HCV mono-infection. Notably, marked restoration of α-diversity was particularly observed in both patient groups achieving sustained virologic response, suggesting therapeutic efficacy in microbiota modulation across infection statuses.^[15]^

Most literature indicates that although there is a difference in species composition before and after DDA treatment, the difference is not statistically significant. At the phylum level, the proportions of Bacteroidetes and Fusobacteria decreased, whereas the proportion of Firmicutes and Verrucomicrobia increased. Furthermore, Bacteroides, Phascolarctobacterium, and Fusobacterium abundance declined at the genus level, while Lachnospira, Faecalibacterium, Oscillospira, and Akkermansia increased. Studies report restored alpha-diversity comparable to healthy controls among patients with mild fibrosis, accompanied by increased beneficial genera (Parabacteroides, Subdoligranulum) and reduced pathogenic Eubacterium abundance.

#### 1.1.2. Long-term effects of DAA on gut microbiota in hepatitis C-infected patients

Wellhöner et al indicated that at SVR48, bacterial diversity and microbial community structure were improved in patients who acquired SVR compared to baseline clinical status. Notably, patients achieving SVR without cirrhosis demonstrated restored microbial diversity. In contrast, cirrhotic SVR patients showed no significant alterations in bacterial profiles before and after direct-acting antiviral therapy. At SVR48, a comparative analysis of cirrhotic versus non-cirrhotic patients revealed taxonomic shifts in 8 bacterial genera. Cirrhosis-associated microbiota exhibited enrichment of Acidaminococcus, Eubacterium, and Lachnospiraceae genera while demonstrating depletion of Citrobacter, Enterobacter, Enterococcus, Megasphaera, and Pseudomonas taxa.^[19]^

However, Ponziani et al followed 12 patients with cirrhosis for up to 1 year. They found significant improvement in alpha-diversity and overall gut microbiome composition changes after HCV eradication. The therapeutic intervention significantly reduced pathobiont populations (Enterococcus, Staphylococcus) and Enterobacteriaceae family members, as demonstrated by post-therapeutic assessment.^[13]^ Besides, Chuaypen et al further evaluated the long-term effects of DAA therapy based on the study, which indicated the short-term impact of DAAs (12 weeks after treatment). Restored alpha-diversity, BCoAT, and lipopolysaccharide-binding protein were observed in HCV patients with F0–F1 fibrosis, with partial restoration in HCV/HIV-coinfected individuals at week 72—treatment modulated microbiota by enriching short-chain fatty acids (SCFA)-producing Blautia, Fusicatenibacter, Subdoligranulum, and Bifidobacterium.^[20]^

### 1.2. Therapeutic prospects of probiotics in chronic hepatitis C disease

It has been reported that the gut microbiota plays a role in the pathogenesis of chronic liver disease because of the anatomical and functional gut–liver connection through the hepatic portal vein system.^[21,22]^ Probiotics, defined as viable microbial organisms exerting beneficial effects through gastrointestinal tract activity, demonstrate significant health-promoting properties.^[23]^ Among these therapeutic agents, *Lactobacillus* and *Bifidobacterium* genera constitute the predominant probiotic taxa, functioning as potent immunomodulatory agents via host–microbe interactions.^[24,25]^ *Lactobacillus acidophilus* (*L acidophilus*) is one of the most important and common intestinal flora, which may change rapidly with intestinal conditions and has been found to increase the cytotoxic activity of natural killer cells.^[26]^

Evidence demonstrates that gut microbial communities serve as critical modulators of the liver–gut axis’s bidirectional communication, mediating hepatoprotective effects by suppressing inflammatory responses, mitigating oxidative stress cascades, and attenuating pathological lipid accumulation in hepatic tissues.^[23]^ Probiotic supplementation in rats ameliorated hepatic pathology, glucose metabolism biomarkers, and gut barrier integrity while restoring microbiota homeostasis and attenuating systemic inflammation, potentially via LPS/TLR4 pathway-mediated immunomodulation.^[27]^ *L acidophilus*-derived antimicrobial peptides isolated in a recent in vitro investigation conducted by Akter et al demonstrated specific bactericidal efficacy against the aquatic pathogen *Aeromonas hydrophila*.^[28]^ Moreover, another experimental study showed that *Bifidobacterium*-containing synbiotics conferred rotavirus protection in rat models via upregulated TNF-α, IL-4, IFN-γ, and TLR2 expression.^[29]^ Therefore, it has been reported that probiotic therapy may be a promising target for preventing and controlling liver disease.

Ashour et al assessed patients with varying hepatitis C virus viremia at pre-fibrosis stages (F0–1), observing higher fecal *L acidophilus* and total lactic acid bacteria in F0–1 versus F > 1 patients; however, no significant correlation emerged between microbial counts and HCV viral load/fibrosis severity, potentially limited by small cohort size.^[30]^ Consistently, Jantararussamee et al demonstrated that a multistrain probiotic formulation (*Lactobacillus casei*, *Lactobacillus paracasei*, *Weissella confusa*) exerted hepatoprotective effects in fibrotic rat models, attenuating hepatic oxidative stress, inflammatory responses, and fibrogenesis.^[31]^ Besides, longitudinal analyses revealed an inverse correlation between hepatitis C virus viremia persistence and intestinal microbial biomass, with elevated sustained viral loads corresponding to progressive enteric dysbiosis. Allam et al demonstrated that probiotic supplementation (*L acidophilus* and *Bifidobacterium* spp.) elevated immune cell counts in 20 chronic HCV patients, correlating with a 25% improvement in pegylated IFN-α/ribavirin therapeutic efficacy.^[32]^

### 1.3. Liver–gut axis and chronic hepatitis C

Dysregulation of gut ecology is common in patients with various chronic liver diseases, especially those with advanced liver fibrosis or cirrhosis,^[33,34]^ but its causes remain elusive. One hypothesis is that the etiology of liver disease, such as viral infection, alcohol exposure, or metabolic disorders, can alter the gut microbiota on its own. Therefore, optimal investigation of HCV pathogenesis requires acute-phase gut microbiome sampling to establish causality between viral acquisition and dysbiosis, though methodological constraints complicate implementation. Most acute HCV infection patients lack clinical symptoms. An alternative pathogenic paradigm posits that gut microbial dysbiosis potentiates portal endotoxin translocation, thereby driving hepatic inflammatory-fibrotic cascades through microbiota-derived pathogen-associated molecular patterns.^[17]^ Alternatively, etiologic factors are not themselves the cause of ecological dysregulation. Rather, structural and physiologic disturbances caused by long-term hepatic dysfunction (e.g., increased intestinal permeability, visceral vasodilatation, or impaired antimicrobial immunity) drive ecological dysregulation, leading to a vicious cycle.^[33]^ Oh et al established that conserved gut microbial biomarkers diagnostically distinguish cirrhotic patients across transcontinental cohorts, demonstrating robustness against interindividual variations in etiological drivers, host genetics, and environmental determinants.^[35]^ Given the ubiquity of this microbial signature, a common pathophysiologic mechanism rather than an etiology-specific one is more likely to explain the dysregulation of gut ecology in advanced liver diseases.

From analyzing metabolomics, Takako Inoue et al reported for the first time that hepatitis C virus infection is associated with abnormal bile acid metabolism in the gut–microbe–hepatic axis.^[36]^ The results show that patients with hepatitis C, even in the early stages of the disease, display a bile acid profile that differs from that of healthy individuals, with a significant decrease in fecal deoxycholic acid and the dominance of lithocholic acid or ursodeoxycholic acid. The team concluded that hepatitis C alters the distribution of bile acids in the gut in association with imbalances in bile acid biosynthesis that differ from the pattern in NAFLD. These imbalances appear to drive disease progression through the gut–microbe–liver axis. At the same time, however, researchers highlighted that existing literature predominantly employs cross-sectional designs focused on acute/chronic HCV infection phases, lacking longitudinal analyses of pre-/postinfection microbial dynamics. The characterization of the microbiome and bile acid metabolism after HCV elimination, especially in patients with cirrhosis, still requires more studies to be further elucidated. In addition, the studies mentioned above have also shown that effective DAA can increase the abundance of beneficial bacteria, especially SCFA-producing bacteria. The pathogenesis of NAFLD is also related to SCFA,^[37]^ and perhaps some research ideas on hepatitis C can be derived from their studies, but further studies are still needed.

## 2. Discussion

In this review, we summarized the changes in gut microbiota in CHC patients before and after DAA treatment, put forward our views on applying probiotics in CHC, and further analyzed the relationship between gut microbiota and CHC.

Many studies have shown that DAA treatment does not change the diversity of intestinal flora in patients in the short to medium term. Despite the differences in the gut microbiota composition, the differences were insignificant. The heterogeneity of these results across studies likely stems from limited cohort sizes and unaccounted confounding variables encompassing host genetics, dietary patterns, immune status, and environmental microbiota exposure^[38]^; these factors are hard to control for in a clinical study. Their findings further suggested that HCV infection failed to perturb gut microbiota profiles acutely within 12 to 24 weeks. Based on extended longitudinal monitoring, sustained viral clearance restored microbial architecture and short-chain fatty acid synthesis, correlating with ameliorated enteric microbial translocation—particularly in F0–F1 fibrosis patients. These data also reassured that DAA treatment did not exert substantiative effects on the intestinal commensals. However, whether gut microbiota improves with follow-up time remains to be further studied.

Previous biome studies have shown that the diversity of fecal microbial communities is lower in patients with CHC than in controls. This change is more pronounced in the presence of liver cirrhosis.^[8]^ This low diversity is thought to result from hepatitis C virus infection disrupting the dynamic balance of gut microbes and immune adaptation to chronic infection conditions.^[21]^ Although the cause of intestinal flora disorders in CHC is not well understood, early treatment with DAAs is recommended for any HCV-infected person to prevent complications other than liver disease. In addition to antiviral therapy, as early as possible, we should promptly use probiotics and other drugs that regulate the gut, especially when hepatitis is low in fibrosis and viral load. This has a great impact on the progression and prognosis of the disease. However, further studies, including a larger population sample as well as longitudinal studies, are required to determine the specific usage and dosage.

For patients with chronic hepatitis or even cirrhosis, whether gut microbiota diversity is restored is still controversial, and most of the study results showed that the improvement of gut microbiota was not obvious for patients with high fibrosis, however, how to define the follow-up time to exclude the effect brought by the recovery of liver function is a question to be considered. In addition, the intestinal microecological diversity did not change much after DAA eradication, suggesting that DAA treatment may not have a substantial effect on intestinal microecology and also suggesting that the possible etiologic factors may have less impact on intestinal microecology and that it is more the etiologic factors that cause the abnormalities in liver function that cause the changes in intestinal microecology, which promotes the progression of CHC, and ultimately creates a vicious circle.

Current studies suffer from small sample sizes, definition of study follow-up times, and control of other influencing factors. In addition, there are fewer metabolomic studies of gut flora in hepatitis C, and there is a lack of many original articles to elucidate the mechanisms involved further. It is worth noting that more studies are needed to analyze the changes in intestinal microecology before and after the use of antiviral drugs in special populations, such as those with dual infections and co-infections. The search strategy is attached as Supplemental Content, Supplemental Digital Content, https://links.lww.com/MD/O763 after the article.

## 3. Conclusion

DAA treatment had no substantial effect on gut commensal bacteria. Cure of hepatitis C may restore gut microbiota composition and SCFA production, especially in HCV mono-infected patients with low-grade fibrosis, although early changes in gut microbiota are not significant. In addition to antiviral therapy, as early as possible, we should promptly use probiotics and other drugs that regulate the gut, especially when hepatitis is low in fibrosis and viral load, which would help prevent long-term hepatic and extrahepatic complications associated with gut flora dysbiosis.

## Author contributions

**Conceptualization:** Hui Li.

**Methodology:** Hui Li.

**Data curation and software:** Yang Liu.

**Investigation:** Hui Li, Yang Liu.

**New software and visualization:** Hui Li, Yang Liu.

**Writing – original draft:** Yang Liu.

**Writing – review & editing:** Hui Li, Ming Yu Lai.

**Resources and project administration:** Hui Li, Ming Yu Lai.

## Supplementary Material


