# Supplementary material for: Intestinal flora of hepatitis C after direct antiviral drug therapy: A review
Source: Medicine (Baltimore). 2025 Aug 1;104(31):e42301. doi: 10.1097/MD.0000000000042301 (PMC12324025; doi:10.1097/MD.0000000000042301)
Supplement: Supplementary file 1 [file medi-104-e42301-s001.docx]

Supplementary content: The search strategy in the paper.

References were searched in PubMed, Web of Science, and Embase to analyze the change in intestinal microecology in hepatitis C after direct antiviral drug therapy from the time of

library construction to March 2024. Most of the references are from the last five years. English search terms included (1)“hepatitis c” OR “chronic hepatitis c” OR “hepatitis c, chronic” OR “'parenterally-transmitted non-a, non-b hepatitis”; (2) “gastrointestinal microbiomes” OR “gut microbiome” OR “gut microflora” et al; (3) “antiviral agents” OR “agents, antiviral” OR “antivirals” OR “antiviral” et al.

Pubmed

#1 ("Hepatitis C"[Mesh]) OR (((((((Hepatitis, Viral, Non-A, Non-B, Parenterally-Transmitted) OR (Parenterally-Transmitted Non-A, Non-B Hepatitis)) OR (Parenterally Transmitted Non A, Non B Hepatitis)) OR (PT-NANBH)) OR (Hepatitis C)) OR (Hepatitis C, Chronic)) OR (Chronic Hepatitis C))

#2 ("Gastrointestinal Microbiome"[Mesh]) OR ((((((((((((((((((((((((((((((((((((((Gastrointestinal Microbiome) OR (Gastrointestinal Microbiomes)) OR (Microbiome, Gastrointestinal)) OR (Gastrointestinal Microbial Community)) OR (Gastrointestinal Microbial Communities)) OR (Microbial Community, Gastrointestinal)) OR (Gut Microbiome)) OR (Gut Microbiomes)) OR (Microbiome, Gut)) OR (Gut Microflora)) OR (Microflora, Gut)) OR (Gastrointestinal Microflora)) OR (Microflora, Gastrointestinal)) OR (Gastrointestinal Flora)) OR (Flora, Gastrointestinal)) OR (Gut Flora)) OR (Flora, Gut)) OR (Gastrointestinal Microbiota)) OR (Gastrointestinal Microbiotas)) OR (Microbiota, Gastrointestinal)) OR (Gut Microbiota)) OR (Gut Microbiotas)) OR (Microbiota, Gut)) OR (Intestinal Microbiome)) OR (Intestinal Microbiomes)) OR (Microbiome, Intestinal)) OR (Intestinal Flora)) OR (Flora, Intestinal)) OR (Intestinal Microbiota)) OR (Intestinal Microbiotas)) OR (Microbiota, Intestinal)) OR (Intestinal Microflora)) OR (Microflora, Intestinal)) OR (Enteric Bacteria)) OR (Bacteria, Enteric)) OR (Gastric Microbiome)) OR (Gastric Microbiomes)) OR (Microbiome, Gastric))

#3 ("Antiviral Agents"[Mesh]) OR (((((((((((Antiviral Agents) OR (Antiviral Agents)) OR (Agents, Antiviral)) OR (Antivirals)) OR (Antiviral)) OR (Antiviral Drugs)) OR (Drugs, Antiviral)) OR (Antiviral Drug)) OR (Drug, Antiviral)) OR (Antiviral Agent)) OR (Agent, Antiviral))

#4 #1and#2and#3

Web of Science

(TS=(Gastrointestinal Microbiome) OR AB=(Gastrointestinal Microbiome OR Gastrointestinal Microbiomes OR Microbiome, Gastrointestinal OR Gut Microbiome OR Gut Microbiomes OR Microbiome, Gut OR Gut Microflora OR Microflora, Gut OR Gut Microbiota OR Gut Microbiotas OR Microbiota, Gut OR Gastrointestinal Flora OR Flora, Gastrointestinal OR Gut Flora OR Flora, Gut OR Gastrointestinal Microbiota OR Gastrointestinal Microbiotas OR Microbiota, Gastrointestinal OR Gastrointestinal Microbial Community OR Gastrointestinal Microbial Communities OR Microbial Community, Gastrointestinal OR Gastrointestinal Microflora OR Microflora, Gastrointestinal OR Gastric Microbiome OR Gastric Microbiomes OR Microbiome, Gastric OR Intestinal Microbiome OR Intestinal Microbiomes OR Microbiome, Intestinal OR Intestinal Microbiota OR Intestinal Microbiotas OR Microbiota, Intestinal OR Intestinal Microflora OR Microflora, Intestinal OR Intestinal Flora OR Flora, Intestinal OR Enteric Bacteria OR Bacteria, Enteric)) AND (TS=(Hepatitis C) OR AB=(Hepatitis C OR Chronic Hepatitis C OR Hepatitis C, Chronic)) AND (TS=(Antiviral Agents) OR AB=(Antiviral Agents OR Antiviral Agents OR Agents, Antiviral OR Antivirals OR Antiviral OR Antiviral Drugs OR Drugs, Antiviral OR Antiviral Drug OR Drug, Antiviral OR Antiviral Agent OR Agent, Antiviral))

Embase:

#1 'chronic hepatitis c':ab,ti OR 'hepatitis c, chronic':ab,ti OR 'hepatitis c':ab,ti OR 'Hepatitis, Viral, Non-A, Non-B, Parenterally-Transmitted':ab,ti OR 'Parenterally-Transmitted Non-A, Non-B Hepatitis':ab,ti OR 'Parenterally Transmitted Non A, Non B Hepatitis':ab,ti OR 'PT-NANBH':ab,ti

#2 'gastrointestinal microbiome':ab,ti OR 'gastrointestinal microbiomes':ab,ti OR 'microbiome, gastrointestinal':ab,ti OR 'gut microbiome':ab,ti OR 'gut microbiomes':ab,ti OR 'microbiome, gut':ab,ti OR 'gut microflora':ab,ti OR 'microflora, gut':ab,ti OR 'gut microbiota':ab,ti OR 'gut microbiotas':ab,ti OR 'microbiota, gut':ab,ti OR 'gastrointestinal flora':ab,ti OR 'flora, gastrointestinal':ab,ti OR 'gut flora':ab,ti OR 'flora, gut':ab,ti OR 'gastrointestinal microbiota':ab,ti OR 'gastrointestinal microbiotas':ab,ti OR 'microbiota, gastrointestinal':ab,ti OR 'gastrointestinal microbial community':ab,ti OR 'gastrointestinal microbial communities':ab,ti OR 'microbial community, gastrointestinal':ab,ti OR 'gastrointestinal microflora':ab,ti OR 'microflora, gastrointestinal':ab,ti OR 'gastric microbiome':ab,ti OR 'gastric microbiomes':ab,ti OR 'microbiome, gastric':ab,ti OR 'intestinal microbiome':ab,ti OR 'intestinal microbiomes':ab,ti OR 'microbiome, intestinal':ab,ti OR 'intestinal microbiota':ab,ti OR 'intestinal microbiotas':ab,ti OR 'microbiota, intestinal':ab,ti OR 'intestinal microflora':ab,ti OR 'microflora, intestinal':ab,ti OR 'intestinal flora':ab,ti OR 'flora, intestinal':ab,ti OR 'enteric bacteria':ab,ti OR 'bacteria, enteric':ab,ti

#3 'Antiviral Agents':ab,ti OR 'Agents, Antiviral':ab,ti OR 'Antivirals':ab,ti OR 'Antiviral':ab,ti OR 'Antiviral Drugs':ab,ti OR 'Drugs, Antiviral':ab,ti OR 'Antiviral Drug':ab,ti OR 'Drug, Antiviral':ab,ti OR 'Antiviral Agent':ab,ti OR 'Agent, Antiviral':ab,ti

#4 #1and #2and #3
